# Intracellular delivery of peptides via association with ubiquitin or SUMO-1 coupled to protein transduction domains

**DOI:** 10.1186/1472-6750-8-24

**Published:** 2008-02-29

**Authors:** Anne-Laure Vitte, Pierre Jalinot

**Affiliations:** 1 LBMC, UMR5239 CNRS – ENS de Lyon, IFR 128 Biosciences Lyon Gerland 46 Allée d'Italie, 69364 Lyon cedex 07, France

## Abstract

**Background:**

We previously developed small hybrid proteins consisting of SUMO-1 linked to an heptapeptide fused to the Tat protein transduction domain (PTD). The heptapeptide motif was selected from a library of random sequences to specifically bind HIV-1 regulatory proteins Tat or Rev. These constructs, named SHP, are able to enter primary lymphocytes and some of them inhibit HIV-1 replication. Considering these positive results and other data from the literature, we further tested the ability of ubiquitin or SUMO-1 linked to various PTD at their N-terminus to deliver within cells proteins or peptides fused downstream of their diglycine motif. In this system it is expected that the intracellular ubiquitin or SUMO-1 hydrolases cleave the PTD-Ub or PTD-SUMO-1 modules from the cargo polypeptide, thereby allowing its delivery under an unmodified form.

**Results:**

Several bacterial expression vectors have been constructed to produce modular proteins containing from the N- to the C-terminus: the FLAG epitope, a cleavage site for a protease, a PTD, human ubiquitin or SUMO-1, and either GFP or the HA epitope. Nine different PTDs were tested, including the Tat basic domain, wild type or with various mutations, and stretches of arginine or lysine. It was observed that some of these PTDs, mainly the Tat PTD and seven or nine residues long polyarginine motifs, caused association of the hybrid proteins with cells, but none of these constructs were delivered to the cytosol. This conclusion was derived from biochemical and immunofluorescence studies, and also from the fact that free cargo protein resulting from cleavage by proteases after ubiquitin or SUMO-1 was never observed. However, in agreement with our previous observations, mutation of the diglycine motif into alanine-arginine, as in the SHP constructs, allows cytosol entry demonstrated by immunofluorescence observations on living cells and by cell fractionation analyses. This process results from a non-endocytic pathway.

**Conclusion:**

Our observations indicate that fusion of SUMO-1 to a peptide-PTD module allows generation of a stable hybrid protein that is easily produced in bacteria and which efficiently enters into cells but this property necessitates mutation of the diglycine motif at the end of SUMO-1, thereby impairing delivery of the peptide alone.

## Background

The rapid progress in the understanding of protein networks underlying biological functions, as well as of the specific roles played by particular polypeptides in human pathologies such as cancer, has fuelled the search for means to deliver peptides or proteins into cells within a therapeutic perspective. Exciting developments originated from previous studies on the viral transactivator Tat, as well as the antennapedia transcription factor [1-3]. Characterization of the capacity of these proteins to enter cells led to the mapping of peptidic domains of limited size responsible for this property which turned out to be transferable by linkage to various peptides or proteins [4-6]; for a recent review see [7]). These so-called protein transduction domains (PTD) or cell-penetrating peptides raised the possibility of delivering an exogenous protein component into cells. This has been established for many different proteins or peptides ex vivo and has also been shown to work in the whole animal [8]. However, several recent studies have raised doubts concerning the veritable capacity of such hybrid proteins to enter cells [7,9-12]. For immunofluorescence studies in particular, the fixation step has been shown to cause possible artefacts. In some cases reported cellular entry is therefore questionable, but in others the observed biological effects are difficult to explain without authentic cellular delivery [13-15]. The exact molecular mechanism that allows penetration within cells is also confusing. This property has been shown in some instances to be independent of energy consumption but in others to involve various forms of endocytosis [7,12,16-19]. A detailed study with a hybrid TAT-CRE construct has shown that cellular entry was achieved through macropinocytosis [15]. From the published data it appears that the exact mechanism involved depends on the precise nature of the protein and of the PTD. The cell type is also probably important.

A potential problem with peptides or proteins to be delivered into cells is their instability. Association with a folded stable domain can increase this stability. Ubiquitin or members of this protein family can be interesting in this perspective. Several expression systems in bacteria or eukaryotes have benefited from this property favouring the production of otherwise poorly-expressed proteins [20-24]. Indeed, fusion of ubiquitin to their N-terminus can allow the production of such proteins. This is also the case with SUMO-1. In addition, association with ubiquitin or SUMO-1 allows easy cleavage after the diglycine motif which terminates the protein. This possibility has been used in particular for the experimental system which allowed characterization of the N-end rule which states that the stability of a protein depends on the nature of its amino-terminal residue [25].

Considering these notions, we designed a system to deliver proteins into cells without any addition by creating fusions with PTD-ubiquitin or PTD-SUMO-1 hybrids. Although the system allows efficient expression in bacteria and easy purification, it appeared that these hybrid proteins do not allow efficient delivery into cells. By contrast, mutation of the diglycine motif and association with a peptide motif linked to the Tat or poly arginine PTD permits efficient entry and the mechanism sustaining this entry is energy-independent.

## Results and discussion

### Design of fusion proteins with the capability of delivering a given protein

**Figure 1:**
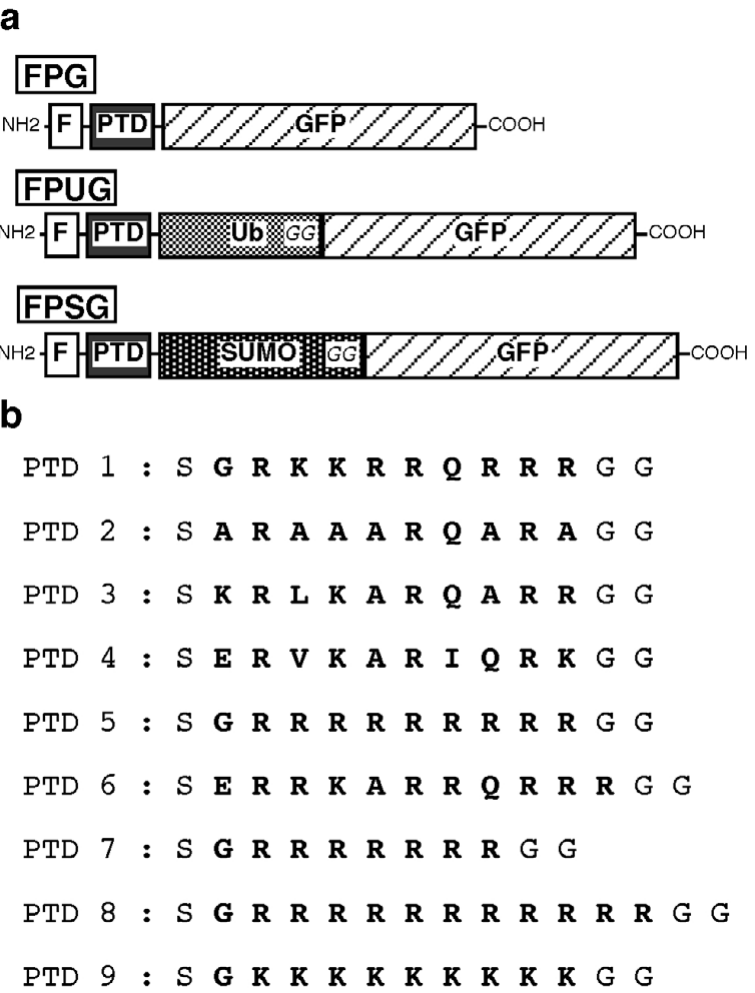
**Fusion proteins associating GFP with various protein transduction domains and either ubiquitin or SUMO-1.(a) **Schematic representation of the different types of fusion proteins. Vectors were constructed to express GFP as a fusion with the FLAG epitope (F) and with a protein transduction domain (PTD) giving proteins FPG. For other constructs, ubiquitin (Ub) or SUMO-1 (SUMO) was inserted between the PTD and the GFP fragments, so as to allow delivery of GFP, through cleavage after the diglycine motif (GG) present within both ubiquitin and SUMO-1. **(b) **Different oligonucleotides were designed and annealed, so as to generate 9 different possible PTDs. These sequences were inserted after those coding for the FLAG epitope. These PTDs corresponded to the basic domain of the HIV-1 Tat protein (PTD1), to an Ala-rich derivative (PTD2), to poly-arginine motifs (PTD5, PTD7, PTD8) and to a polylysine motif (PTD9). PTD3, 4 and 6 were derived from the alpha-helical domain present within ubiquitin.

Association of various motifs with a peptide or protein has been shown to trigger cell entry from the extracellular milieu. Well-characterized examples of such motifs are the Tat basic domain, stretches of arginine, and part of the antennapedia transcription factor [4-6,26]. However, these protein motifs are generally covalently linked to the peptide or protein to be delivered, and hence remain associated with it permanently, thereby potentially altering their properties. In particular the Tat basic domain or polyarginine motif confers intranuclear localisation or association with nucleic acids, a feature which might not be wanted. To avoid this, we tested a system which associates the protein with a module composed of the FLAG epitope, a PTD and either ubiquitin or SUMO-1 (Figure 1a). The diglycine motif of these small proteins is directly linked to the first amino acid of the protein to be delivered, which in our constructions was GFP, taken as a model polypeptide. It was anticipated that within cells ubiquitin or SUMO-1 proteases would cleave the fusion protein after the diglycine motif, thereby releasing GFP starting at the first amino acid. This is known to occur in particular for the ubiquitin ribosomal protein fusions encoded by several cellular genes [27]. The constructs were designed to allow easy insertion of various PTDs after the FLAG epitope (Figure 1a). These two elements were separated by a cleavage site for the Prescission protease, thereby allowing removal of the FLAG epitope if necessary. The efficiency of nine different PTDs was tested using these constructs (Figure 1b). PTD 1 corresponds to the HIV-1 TAT basic domain. PTD 2 was defined by Ho et al. (2001) as an efficient derivative of the former. These authors proposed that strengthening of the α-helical structure of the motif reinforces its transduction properties [28]. With the idea of introducing these modifications in ubiquitin itself at a later stage, PTD 3, 4 and 6 were designed from the α-helix present in the ubiquitin fold with the aim of increasing its α-helical nature, as well as its content in basic residues to confer transduction properties. PTD 5, 7 and 8 are stretches of 9, 7 and 11 arginines, respectively. Finally, PTD 9 corresponds to a stretch of 9 lysines.

**Figure 2:**
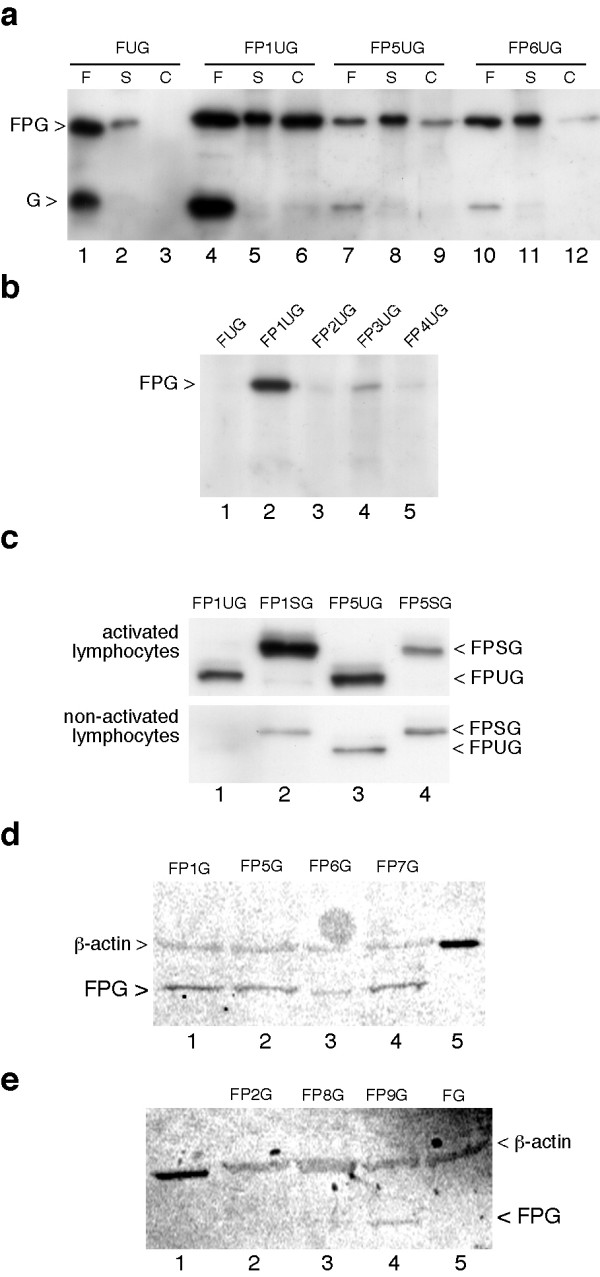
**Association of the various GFP fusion proteins with cells.(a) **Jurkat cells were incubated for 1 h with 0.5 μM of FUG (lanes 1 to 3), FP1UG (lanes 4 to 6), FP5UG (lanes 7 to 9) and FP6UG (lanes 10 to 12). After PBS wash, cells were further incubated in RPMI for 4 h. Cells were then washed again with PBS and after collection by centrifugation were lysed in protein loading buffer. Cell extracts (C), together with aliquots of the purified protein (F) and of the first incubation supernatant (S), were loaded onto a SDS protein gel. Immunoblot analysis was carried out using an antibody to GFP. Positions of the signal corresponding to the complete fusion protein (FPG) and of a cleavage product corresponding to GFP (G) are indicated on the left. **(b) **Proteins FUG, FP1UG, FP2UG, FP3UG and FP4UG were tested as described for panel A, except that only cell extracts were analysed by immunoblot. **(c) **Activated or non-activated primary lymphocytes were incubated with 0.5 μM of FP1UG, FP1SG, FP5UG or FP5SG for 2 h. Cells were then washed with PBS and lysed in protein loading buffer. Immunoblot analysis was performed as described for panel A and the positions of the signals corresponding to FPSG and FPUG are indicated on the right. **(d) **and **(e) **Activated lymphocytes were incubated for 1 h with 0.5 μM of FPG fusion proteins as indicated. This was also done with the FG protein which lacks a PTD motif. After incubation with the proteins, cells were washed in PBS and further incubated in RPMI for 4 h. After a PBS wash, cells were lysed in protein loading buffer. Immunoblot analysis was carried out using a mix of antibody to GFP and to β-actin. Detection of this latter protein was performed to control protein loading in each lane. Revelation was performed with a fluorescent secondary antibody. Positions of signals corresponding to β-actin and FPG are indicated.

To examine the efficiency of these various PTDs, the ubiquitin-GFP fusions were expressed in bacteria and affinity-purified using anti-FLAG beads. All these proteins were produced in large amounts and easily purified (A-L Vitte, data not shown). These proteins were then incubated with Jurkat cells which were lysed in SDS buffer and analyzed by immunoblot using an antibody to GFP. As controls, aliquots of purified protein and cell supernatant were also analyzed. With purified proteins a certain amount of cleavage occurred between Ub and GFP and bands were seen both at the position of the complete fusion protein and of GFP (Figure 2a, lanes 1, 4, 7 and 10). Depending on the various PTDs, proteins were observed or not in the cell extracts. This was clearly the case for PTD 1 and 5 (Figure 2a, lanes 6 and 9) and less efficiently for PTDs 6 and 3 (Figure 2a, lane 12 and Figure 2b, lane 4). PTDs 2 and 4 were inactive in this assay (Figure 2b, lanes 3 and 5). The protein was also absent in the cell extract when it did not include a PTD (Figure 2a, lane 3 and Figure 2b, lane 1). Unexpectedly, with active PTDs only the complete fusion protein was observed and no signal was detected at the position of GFP alone (Figure 2a, lanes 6 and 9).

We next tested the efficiency of these PTDs in primary lymphocytes. This was done with the ubiquitin and SUMO-1 GFP fusion proteins bearing the Tat and polyR PTDs. These proteins were detected in lymphocytes lysed in SDS and this association was clearly reinforced when lymphocytes were activated (Figure 2c, compare upper and lower panels). The efficiency of the various PTDs was also tested without the presence of ubiquitin or SUMO-1. In the FPG constructs the PTD is directly upstream of the GFP protein. Similarly to what was observed with the ubiquitin fusion proteins, PTDs 1 and 5 mediated association of GFP with cells (Figure 2d, lanes 1 and 2). PTD 6 was weakly active compared to the Tat and polyR motifs (Figure 2d, lane 3). Interestingly, PTD 7 which corresponds to a stretch of seven arginines was as active as PTD 5 which has nine arginines (Figure 2d, compare lanes 4 and 2), but by contrast PTD 8 with eleven arginines showed no activity (Figure 2e, lane 3). A weak cell association was also detected with PTD 9 which corresponds to nine lysines (Figure 2e, lane 4).

Taken together these results show that the Tat and polyR7 or polyR9 were the most efficient motifs under these conditions. In agreement with previous observations, the polyR motif efficiency requires an optimum size of seven to nine residues and increasing this length leads to loss of the effect [26].

### Ubiquitin and SUMO-1 fusion proteins do not enter cells

**Figure 3:**
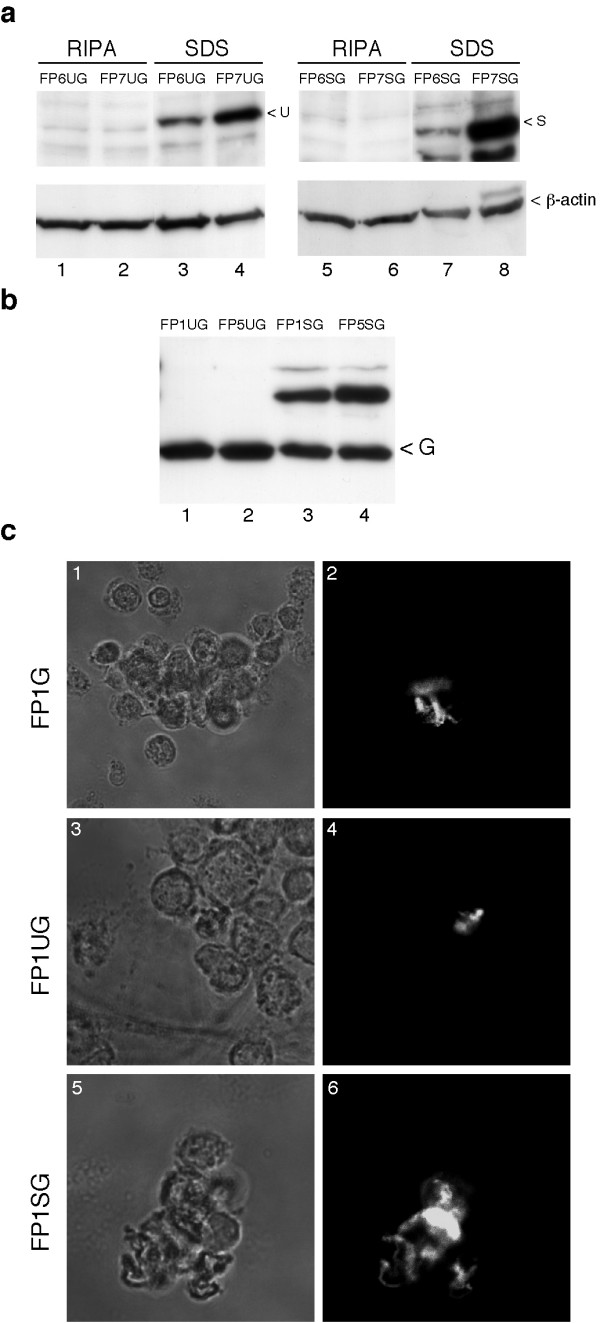
**(a) Activated lymphocytes were incubated for 4 h with 0.5 μM of FP6UG (lanes 1 and 3), FP7UG (lanes 2 and 4), FP6SG (lanes 5 and 7), FP7SG (lanes 6 and 8).** After a PBS wash, cells were lysed either in protein loading buffer (SDS, lanes 3, 4, 7 and 8) or in RIPA buffer (RIPA, lanes 1, 2, 5 and 6). Immunoblot analysis of these extracts was performed with the antibody to GFP (upper panel) or to β-actin (lower panel). Signals corresponding to FPUG (U), FPSG (S) and β-actin are indicated on the right. **(b) **HeLa cells were transfected with 1 μg of plasmid encoding FP1UG, FP1SG, FP5UG or FP5SG. 24 h after transfection, cells were washed with PBS and lysed with RIPA buffer. Samples were loaded onto a 12%-SDS protein gel and an immunoblot was carried out using the antibody to GFP. The position of the signal corresponding to GFP (G) is indicated on the right. **(c) **Activated lymphocytes were incubated for 2 h with 2 μM of FP1G (top panels), FP1UG (middle panels) or FP1SG (bottom panels). Cells were then washed and observed by confocal microscopy. Visualisation was performed either with fluorescence (right panels) or with light transmission (left panels).

Previous observations were carried out lysing cells directly in SDS. Unexpectedly, when lysis was performed in RIPA buffer, these fusion proteins were not detected. Indeed, comparative analysis of both types of extracts clearly showed that fusion proteins with the Tat or polyR PTDs were detected in SDS extracts but not in RIPA lysates (Figure 3a, upper panel). By contrast, equal amounts of β-actin were detected in both extracts (Figure 3a, lower panel). This was true for both ubiquitin and SUMO-1 fusion proteins. This suggests that these proteins are likely to remain with the cellular debris removed by centrifugation during RIPA lysate preparation. Another intriguing point was that complete fusion proteins were detected but no free GFP. To examine whether this was a problem inherent to our constructs or to absence of authentic cell penetration, the sequences coding for the ubiquitin and SUMO-1 GFP fusions associated with the Tat and polyR7 PTDs were cloned into a mammalian expression vector. Cells were transfected with these vectors and then lysed in RIPA buffer. With the ubiquitin fusion a single band was seen at the position of GFP (Figure 3b, lanes 1 and 2). With the SUMO-1 GFP fusion, a band was detected at the position of GFP but another of higher molecular weight was also seen (Figure 3b, lanes 3 and 4). This indicates that processing of these proteins when expressed intracellularly was efficient: completely for ubiquitin and partially for SUMO-1. It also indicates that the resulting GFP is stable. This observation supports the notion that when added from the extracellular milieu these fusions do not veritably enter cells. To analyse this further, we performed immunofluorescence studies. Cells were incubated with the constructs including the Tat PTD directly upstream of GFP or upstream of the ubiquitin or SUMO-1 GFP fusion proteins. For these three proteins only a few discontinuous areas showed GFP staining and comparison with the transmission images showed that they corresponded to debris from dead cells (Figure 3c). Collectively these observations were not in favour of a authentic entry of these fusion proteins into cells. It is more likely that the PTDs allow stable association with the cellular membrane but not veritable delivery of the fusion protein to the cytosol.

**Figure 4:**
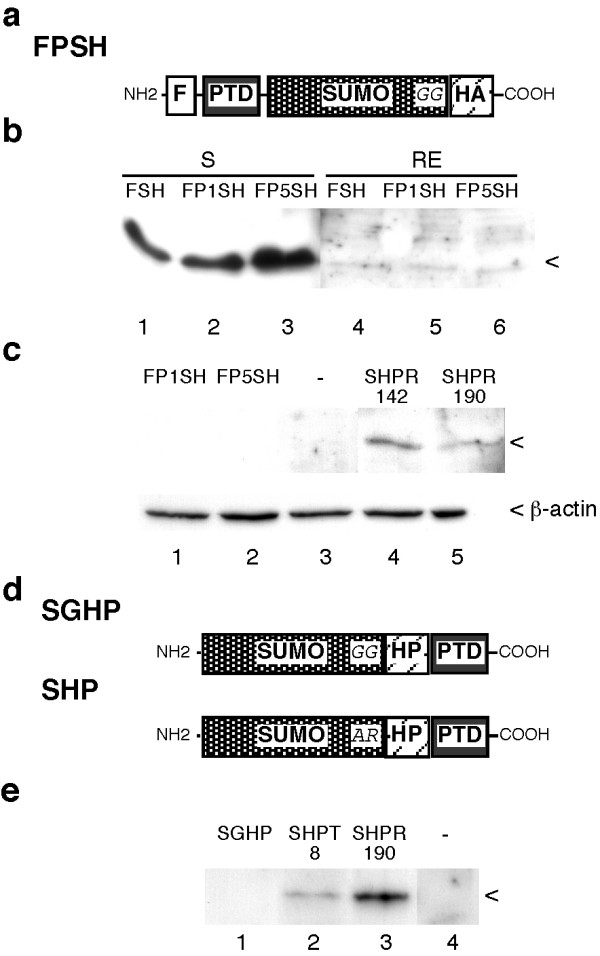
**Negative effect of the SUMO-1 diglycine motif on detection of intracellular fusion proteins.(a) **Schematic representation of fusion protein associating the FLAG-PTD-SUMO-1 module with the HA epitope. Using FPSG vectors, the GFP moiety was deleted and replaced with the HA epitope coding sequence, giving vectors expressing FPSH proteins. **(b) **Activated lymphocytes were incubated for 2 h with 2 μM of the different FPSH proteins, then washed with PBS and lysed in RIPA buffer. Supernatant (S) or RIPA extracts (RE) were analysed by immunoblot using a monoclonal antibody to HA **(c) **Activated lymphocytes were incubated for 2 h without (lane 3) or with 1 μM of either FP1SH (lane 1), FP5SH (lane 2), SHPR142 (lane 4) or SHPR190 (lane 5). Cells were then washed with PBS and lysed with RIPA buffer. Cell extracts were analysed by immunoblot with monoclonal antibodies to SUMO-1 (top panel) and to β-actin (lower panel). Signals corresponding to SUMO-1 fusion proteins and to β-actin are indicated on the right. **(d) **Schematic representation of the SHP and SGHP proteins. Using the vector expressing SHPR-190, the AR motif was restored to the wild type GG sequence occurring at the end of SUMO-1, giving SGHP. **(e) **Activated lymphocytes were incubated with SGHP, SHPT8 or SHPR190 for 2 h and washed in PBS. RIPA extracts were then made and analysed by immunoblot using a monoclonal antibody to SUMO-1.

We then examined if this failure could be due to the size and nature of GFP by replacing this part of the fusion proteins with a small peptide. This was done by placing the HA epitope downstream of SUMO-1 linked either to the Tat or polyR7 PTDs (Figure 4a). Cells lysed in RIPA buffer were analysed by HA and SUMO-1 immunoblot but this did not reveal the presence of the fusion proteins within cells (Figure 4b, lanes 4 to 6). As we have previously shown that fusion proteins corresponding to SUMO-1 associated with a peptide motif together with the Tat PTD efficiently enter cells [14], we compared these proteins with the SUMO-1 HA fusion. As previously reported, SHPR142 and 190 were clearly detected in the RIPA extracts, but this was not the case with the SUMO-1 HA construct (Figure 4c, compare lanes 1 and 2 with lanes 4 and 5). As a clear difference between both types of constructs was that the diglycine motif was mutated in the SHP proteins, thereby impairing their processing, a further study was made to determine whether this mutation could explain the difference. It was found that restoration of the wild type end of SUMO-1 in the SHPR190 construct indeed leads to loss of intracellular entry (Figure. 4d and 4e).

### Veritable cell penetration by the SHPR proteins

**Figure 5:**
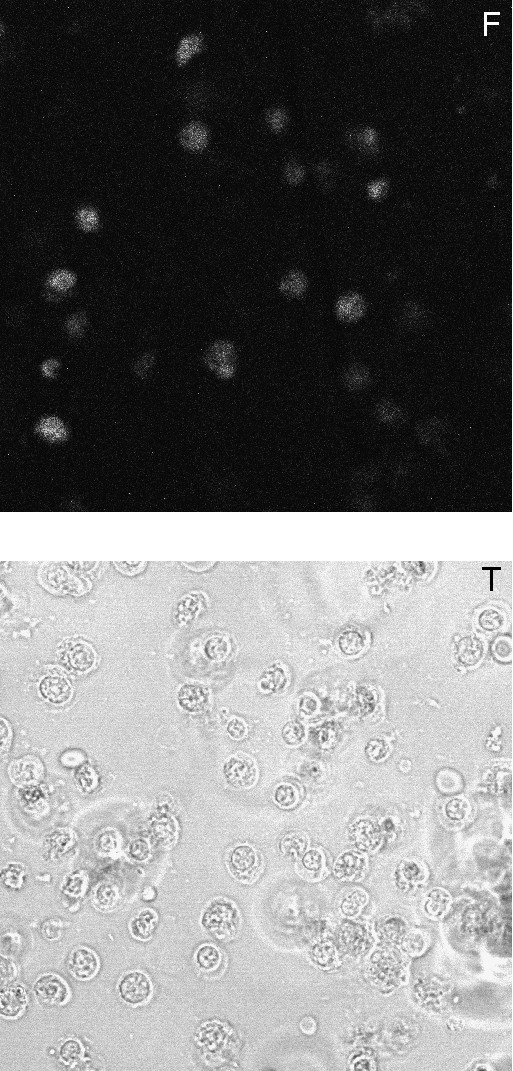
**Activated lymphocytes were incubated for 2 h with 1 μM of SHPR190 labelled with Alexa 488.** Cells were then washed and maintained in HBS buffer for examination by confocal microscopy. Visualisation was performed either by fluorescence (top panel) or by light transmission (bottom panel).

**Figure 6:**
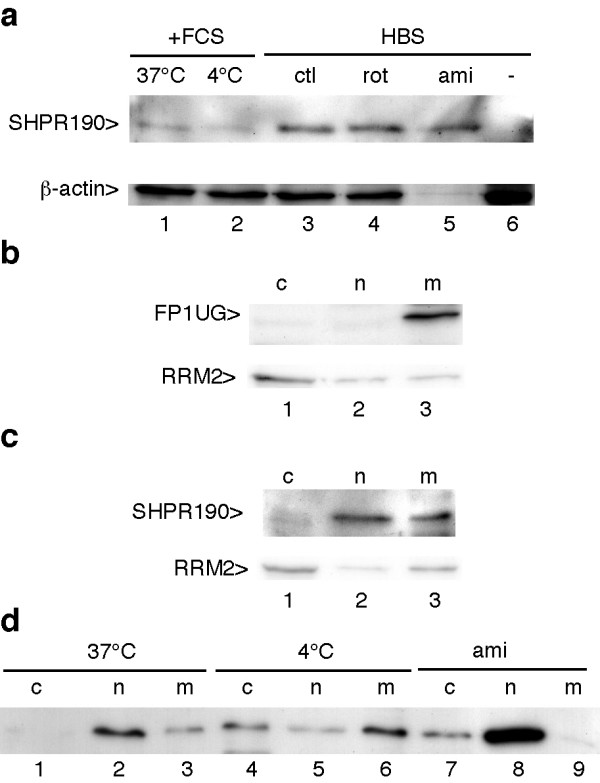
**Nuclear entry of SHPR 190 is energy-independent.(a) **Jurkat cells were cultured either in RPMI 1640 plus FCS at 37°C (lane 1) or 4°C (lane 2), or in HBS buffer containing 10 mM glucose (ctl, lane 3) or 10 mM deoxyglucose plus 1 μM rotenone (rot, lane 4) or 10 mM glucose plus 5 mM amiloride (ami, lane 5) for 30 min. Cells were then incubated with 2 μM of SHPR190 for 2 h, washed in HBS and lysed in RIPA buffer. Cell extracts were analysed by immunoblot using a monoclonal antibody to SUMO-1 (top panel) and to β-actin (bottom panel). **(b) **and **(c) **Jurkat cells were incubated for 2 h either with 4 μM of FP1UG or with 2 μM of SHPR190 in HBS buffer at 37°C. Cells were then washed with HBS and fractionated into cytoplasmic (c, lane 1) nuclear (n, lane 2), and membrane fractions (m, lane 3). Samples of each fraction were analysed by immunoblot using anti-GFP (B) or anti SUMO-1 (C) antibodies (top panels). Cell fractionation was controlled by immunoblot using anti-RRM2 (ribonucleotide reductase peptide M2) antibody (bottom panels). **(d) **Jurkat cells were cultured in HBS buffer either at 37°C (lanes 1 to 3) or 4°C (lanes 4 to 6), or in the presence of 5 mM amiloride (lanes 7 to 9) 30 min before addition of 2 μM of SHPR190. 2 h after incubation, cells were washed in HBS and fractionated into cytoplasmic (m), nuclear (n), and membrane fractions (m). Samples from each fraction were analysed by immunoblot using the monoclonal antibody to SUMO-1.

To strengthen the notion that the SHPR proteins indeed enter cells, immunofluorescence analyses were performed using living cells. The proteins were fluorescently labelled with Alexa 488 and incubated with cells which were washed and maintained in HBS buffer for examination by confocal microscopy. Fluorescence was seen in many cells and comparison with the light transmission image showed that the fluorescent cells had a normal aspect and did not correspond to debris of dead cells (Figure 5). Fluorescence was mainly seen in the nucleus. These observations are in favour of a veritable entry of SHPR within cells. Different studies have reported controversial results about the mechanism of cell entry mediated by PTDs [7]. This process has been reported to involve endocytosis or not. A precise analysis conducted with a CRE-Tat PTD fusion and a reporter gene the expression of which depends on CRE expression, showed that macropinocytosis was involved [15]. It is likely that the intervening mechanism depends on the precise nature of the protein or peptide associated with the PTD [12]. It is possible that for small polypeptides, direct passage through the membrane occurs, whereas for larger proteins endocytosis takes place. To better characterize the process intervening in the case of SHPR, we first tested whether cell entry occurs at 4°C. Although reduced as compared to 37°C, a clear band was seen in RIPA extracts of cells incubated at 4°C with SHPR190 (Figure 6a, lane 2, upper panel). We then investigated if macropinocytosis could participate in the process. To this end we tested the effect of rotenone and amiloride. Both treatments were without effect on the cell entry of SHPR190 (Figure 6a, compare lanes 4 and 5 with lane 3, upper panel). For unclear reasons the β-actin signal was decreased by amiloride treatment but equal total amounts of protein were loaded in the various lanes (Figure 6a, lower panel). For the experiment with amiloride and rotenone, cells were incubated with the protein in HBS, not in normal medium. Under these conditions a higher amount of protein was seen in the cell extract as equal protein amounts were analyzed (Figure 6a, compare lanes 1 and 3, upper panel). In agreement with previous observations [15] this is likely due to an inhibitory effect by the serum. We then investigated in which cell compartment the protein was present. Cells incubated either with FP1UG or SHPR190 were separated into cytoplasmic, nuclear and membrane fractions. When the experiment was performed with the ubiquitin fusion protein associated with the Tat PTD, the protein was seen only in the membrane fraction and no signal was seen in the cytoplasmic and nuclear fractions (Figure 6b, upper panel). Analysis of the cytoplasmic RRM2 protein indicated that the fractionation process was correct (Figure 6b, lower panel). By contrast, when the experiment was done with SHPR190, the protein was mainly seen in the nuclear fraction (Figure 6c, upper panel lane 2), in agreement with the immunofluorescence observations, and a signal was also seen in the membrane fraction. We then tested how reduced temperature or amiloride treatment affects compartmentalization of the protein. Incubation of the cells at 4°C reduced the presence of the protein in the nuclear fraction but it was then detected in the cytoplasm, probably as a result of reduced nuclear import (Figure 6d, lanes 4 to 6). Amiloride treatment did not impair the presence of the protein in the nucleus and even stimulated it (Figure 6d, lane 8). Interestingly, no more protein was seen in the membrane fraction and a low amount was then seen in the cytoplasmic fraction. Considering this effect it is possible that amiloride treatment leads, for unclear reasons, to the release of the protein fraction associated with membranes, thereby increasing its concentration in cytoplasm and nucleus. These data indicate that cell entry of the SHPR protein is energy-independent and does not involve macropinocytosis, a process which requires functional Na^+^/H^+ ^exchange that is inhibited by amiloride [15].

Taken together these observations firmly establish that SHPR proteins are able to penetrate within cells via a mechanism that is energy-independent. Hence this is likely to occur by direct passage through the cellular membrane. The intracellular SUMO-1-peptide fusion is mostly nuclear, probably as consequence of the nucleic acid binding properties of the Tat PTD but also possibly due to the SUMO-1 domain. Indeed, it has been reported for several proteins that SUMO-1 addition triggers nuclear entry. This indicates that our system is probably appropriate for targeting nuclear proteins but not cytoplasmic factors. In the nucleus the SHPs show a diffuse localization. These observations are in agreement with the biological effect of SHPR142 and SHPR190 which are able to block replication of HIV-1 in primary lymphocytes or macrophages when added to the culture medium [14]. The results of the fractionation experiments also support the previous conclusion that the ubiquitin GFP proteins are unable to penetrate within the intracellular milieu. This is in agreement with a previously published report that similar ubiquitin-peptide and ubiquitin-protein constructs are not able to enter the cytosol [29]. However, these authors interestingly showed that dendritic cells were able to uptake and process such hybrid proteins, at least to some extent. Hence, in future studies it will be interesting to test if our ubiquitin or SUMO-1 hybrids can be processed in such cells. An intriguing aspect of our observations is the role of the diglycine motif. Indeed, its presence seems to have a strong negative effect on detection of the SUMO-1-peptide hybrid in cells. As the presence of ubiquitin proteases in the cellular membrane has been reported, it is possible that cleavage after the C-terminal diglycine motif occur simultaneously to crossing of the membrane. However, we did not observe cleavage products of our hybrid proteins, even under conditions of proteasome activity blockage [30,31]. It remains possible that after cleavage both parts are routed towards endosomes and further degraded in lysosomes. Structural studies have established the importance of the diglycine motif in the interaction of SUMO-1 or ubiquitin with C-terminal hydrolases or isopeptidase [32]. Hence, an expected effect of the mutation of this sequence to alanine-arginine is loss of interaction with these enzymes. It is possible that this event explains the efficient capacity of the SHP proteins to enter cells.

## Conclusion

The results presented in this report clarify the mechanism of the cellular entry of SUMO-1-peptide-PTD constructs and confirm that these constructs can efficiently deliver a peptide into cells. Unexpectedly, they show that it is important to block the cleavage that normally occurs at the junction of SUMO-1 and the peptide by mutating the diglycine motif. These constructs which can be easily produced in bacteria potentially offer an interesting means of delivering a peptide able to act as an agonist or antagonist with respect to a pivotal cellular protein.

## Methods

### Constructs

As a first step both 5'-CATGGGCGATTATAAAGATGACGATAAAGGCGGTCA-3' and 5'-TATGACCGCCTTTATCGTCATCTTTATAATCGCC-3' oligonucleotides were annealed and inserted between the Nco I and Nde I restriction sites of vector pET15b giving vector pET-FLAG. The ubiquitin coding sequence was amplified using the following sense and antisense primers: 5'-GAAGATCTCATATGGGCGGTACCCAAATCTTCGTGAAAACCC-3', 5'-GAAGATCTGCGGCCGCCCGGGATCCATACCACCTCTCAGACGC-3'. The amplified DNA fragment was digested by the Nde I and BamH I restriction enzymes and inserted between Nde I and BamH I restriction sites of pET-FLAG, generating vector pET-FU. The GFP coding sequence was obtained from vector pEGFP-1 (Clontech) by Not I – BamH I digestion and was inserted between the Not I and BamH I restriction sites of vector pET-FUG. The various PTDs were included in pET-FUG by annealing appropriate oligonucleotides and inserting them between the NdeI and KpnI restriction sites of pET-FUG, giving vectors pET-FPUG. In some of these latter vectors the sequence encoding ubiquitin was replaced by that of SUMO-1 by digesting pET-FPUG by Kpn I and BamH I. The SUMO-I sequence was obtained by PCR amplification and inserted between these two restriction sites, giving vectors pET-FPSG. Replacement of the GFP coding sequence by that of the HA epitope was made by digesting vectors pET-FPUG and pET-FPSG by BamH I and Not I and inserting annealed sense and antisense following oligonucleotides: 5'-GATCCTGTCTACCCATACGACGTCCCAGACTACGCTGGTAAGTAAGC-3'; 5'-GGCCGCTTACTTACCAGCGTAGTCTGGGACGTCGTATGGGTAGACAG-3'.

### Protein production in bacteria

BL21(DE3)codon+ E. coli were transformed with plasmids encoding the different fusion proteins. Transformants were selected on LB plates containing ampicillin and colonies were then grown overnight at 37°C in LB broth supplemented with 100 mg/ml ampicillin with shaking at 250 rpm. The overnight culture was diluted 25-fold with fresh LB medium complemented with ampicillin and cultured at 37°C until an OD at 600 nm of 0.9 was reached. Protein expression was then induced by addition of 1 mM IPTG (isopropyl-1-thio-β-D-galactopyranoside, Uptima) and incubation at 24°C for 3 h. Bacteria were collected by centrifugation and lysed by sonication in a buffer containing 20 mM NaCl, 0.1 M Tris-HCl pH 7.4, 10 mM MgCl2, lysozyme, endonuclease and antiprotease agents (Complete, Roche). After removal of cell debris by centrifugation, the extract was dialysed in TBS (20 mM Tris pH 7.6, 137 mM NaCl,) and loaded onto a M2 Anti-FLAG column (anti-FLAG M2 Affinity Gel, Sigma). The column was washed first with TBS supplemented with 200 mM NaCl and then with TBS. The fusion proteins were eluted by competition with 100 μg/ml of FLAG peptide diluted in TBS, followed by washing with TBS. The fusion protein fractions were then combined and dialysed in PBS (Phosphate-Buffered-Saline) supplemented with 0.5 mM β-mercaptoethanol. Following dialysis, proteins were sterilized by filtration through a 0.025 μm membrane. All purified fusion proteins were dissolved in PBS containing 0.5 mM β-mercaptoethanol then aliquoted and stored at -80°C. Fusion protein purity was checked by 12% SDS-PAGE followed by staining with Coomassie Brilliant Blue. Protein concentrations were determined by densitometry analysis using bovine serum albumin (BSA) as the standard ("Bradford test", Biorad).

### Cell culture

Cells were incubated at 37°C in a 5% CO2-humidified atmosphere. HeLa cells were cultured in Dulbecco's modified Eagle's medium and Jurkat cells in RPMI 1640 medium supplemented with 5 and 10% foetal calf serum, respectively. Human peripheral blood mononuclear cells (PBMC) were isolated from the blood of healthy donors using Ficoll density gradients. Activation of the lymphocytes was performed by incubating the cells with 1 mg/ml phytohemagglutinin (PHA) and 20 IU/ml IL-2 for 48 h.

### Immunoblot and immunofluorescence

For immunoblot analysis, the samples were loaded onto a 12% SDS-PAGE and proteins were electrotransferred on a PVDF membrane (Amersham), which was then blocked with PBS Tween 0.1% supplemented with 5% dry milk. Membranes were probed with either mouse monoclonal anti-FLAG M2 antibody (Sigma, dilution 1:1000 in PBS Tween 0.1%), a mouse monoclonal anti-GFP antibody (dilution 1:1000 in PBS Tween 0.1%) or a mouse monoclonal anti-SUMO-1 antibody (Zymed, dilution 1:2000 in PBS Tween 0.1%). Mouse anti-β actin (Sigma, dilution 1:5000) and rabbit polyclonal anti-RRM2 antibody were also used to verify the extracts homogeneity or fractionation purity, respectively. Sheep anti-mouse or anti-rabbit immunoglobulins coupled to peroxidase (Amersham, dilution 1:6000) were used as secondary antibodies. Revelation was performed by chemiluminescence using the ECL or ECL plus reagent (Amersham Biosciences). Alternatively (Figure 2d and 2e), the membrane was incubated with anti-mouse immunoglobulins coupled to cyanin 5 (dilution 1:500), and the signal visualized using a STORM 860 apparatus.

For immunofluorescence, cells treated with the different fusion proteins were washed 3 times with PBS and then placed in HBS culture medium (10 mM Hepes, pH 7.3; 10 mM D-glucose; 135 mM NaCl; 5 mM KCl; 2 mM MgCl_2_; 2 mM CaCl_2_). The cells were then directly loaded onto glass slides coated with 1 mg/ml polylysin by incubation during 5 min followed by a wash with H2O. When GFP fusion proteins were used in the experiment, cells were directly observed for GFP fluorescence by confocal microscopy. Alternatively, SHP proteins, which do not include GFP, were coupled to the Alexa Fluor 488 dye using the Molecular Probes protein labelling kit (A-10235) according to the manufacturer's instructions.

## Abbreviations

GFP: green fluorescent protein; HBS: hepes buffer saline; HIV-1: human immunodeficiency virus type 1; polyR: polyarginine; PTD: protein transduction domain; RIPA: radioimmunoprecipitation assay; SUMO: small ubiquitin-related modifier; SHP: SUMO-1 heptapeptide PTD; SHPR: SUMO-1 heptapeptide PTD Rev; SHPT: SUMO-1 heptapeptide PTD Tat; SDS : sodium dodecyl sulfate; Ub: ubiquitin.

## Authors' contributions

ALV participated in the design of the experiments and carried them out. She was also involved in the writing of the manuscipt. PJ conceived the study and wrote the manuscript. Both authors read and approved the final manuscript.
